# A216 WHEN CANCER CASTS THE FIRST STONE: A CASE REPORT OF GALLBLADDER CANCER PRESENTING AS GALLSTONE ILEUS

**DOI:** 10.1093/jcag/gwab049.215

**Published:** 2022-02-21

**Authors:** J Shelton, M McCall, M Strickland

**Affiliations:** 1 University of Alberta Department of Surgery, Edmonton, AB, Canada; 2 University of Alberta Department of Medicine, Edmonton, AB, Canada

## Abstract

**Background:**

Gallstone ileus is a rare cause of mechanical bowel obstruction. It occurs when a biliary stone passes through a cholecystoenteric fistula and becomes impacted in the bowel lumen, most commonly at the terminal ileum or ileocecal valve.

Gallbladder cancer is rare and often has a poor prognosis. Many patients present with advanced-stage disease and only 10% of patients are candidates for surgical resection. Early research has shown there is an increased incidence of gallbladder cancer among patients with gallstone ileus. To date, this relationship remains poorly understood.

**Aims:**

To present a case of an elderly female whose gallbladder cancer presented after causing a cholecystoenteric fistula and gallstone ileus.

**Methods:**

A retrospective review of a single patient case.

**Results:**

Our patient was a 78-year-old female with a background history of hypertension, iron-deficiency anemia, osteoporosis, and treated bilateral breast cancer. She initially presented with acute cholecystitis, complicated by a concurrent pulmonary embolism. Given her need for systemic anticoagulation, she was treated non-operatively with antibiotics and an outpatient cholecystectomy was planned.

She returned to hospital eight months later reporting two days of nausea and vomiting. A repeat CT scan showed a cholecystoduodenal fistula with a 3.5cm x 2.6cm gallstone impacted 20cm from the ileocecal valve. A laparoscopic-assisted enterolithotomy was performed and her postoperative course was uneventful.

One month later, a CT scan was organized by her family physician to follow-up on incidental liver lesions. While the liver lesions were deemed benign, there was new lobulated soft tissue within the gallbladder, measuring 4.8cm x 4.7cm x 6.2cm, suspicious for a primary gallbladder malignancy. A follow-up MRI confirmed an intraluminal mass with direct invasion into hepatic segment III as well as marked segment III intrahepatic biliary dilatation. Endoscopy to the duodenum and cholecystoduodenal fistula was performed with a biopsy confirming gallbladder adenocarcinoma.

She underwent an open radical cholecystectomy, left hepatic lobectomy, antrectomy, resection of 1st portion of duodenum, and reconstruction with a Roux-en-Y gastrojejunostomy. Final pathology and staging confirmed a pT3pN0M0 adenocarcinoma of the gallbladder with a fistula tract within the cancer extending to the duodenum. She was then referred to a cancer center to complete a six-month course of adjuvant chemotherapy with capecitabine.

**Conclusions:**

Gallstone ileus is a cause of mechanical bowel obstruction and rarely, it can be the first presentation of gallbladder cancer. Gallbladder cancer has a poor prognosis with many patients presenting with late-stage disease. As a result, we recommend the consideration of post-enterolithotomy imaging in select patients to evaluate for evidence of malignancy.

**Funding Agencies:**

None

